# A gut microbiome signature for HIV and metabolic dysfunction-associated steatotic liver disease

**DOI:** 10.3389/fimmu.2023.1297378

**Published:** 2023-12-14

**Authors:** Javier Martínez-Sanz, Alba Talavera-Rodríguez, Jorge Díaz-Álvarez, Marta Rosas Cancio-Suárez, Juan Miguel Rodríguez, Claudio Alba, María Luisa Montes, Rosa Martín-Mateos, Diego Burgos-Santamaría, Santiago Moreno, Sergio Serrano-Villar, Matilde Sánchez-Conde

**Affiliations:** ^1^ Department of Infectious Diseases, Hospital Universitario Ramón y Cajal, Instituto Ramón y Cajal de Investigación Sanitaria (IRYCIS), Madrid, Spain; ^2^ CIBER de Enfermedades Infecciosas (CIBERINFEC), Instituto de Salud Carlos III, Madrid, Spain; ^3^ Universidad Complutense de Madrid (UCM), Madrid, Spain; ^4^ Department of Nutrition and Food Science, Complutense University of Madrid, Madrid, Spain; ^5^ HIV Unit - Internal Medicine Service, Hospital Universitario La Paz, Madrid, Spain; ^6^ Department of Gastroenterology and Hepatology, Metabolic Liver Disease Clinic, Hospital Universitario Ramón y Cajal, Instituto Ramón y Cajal de Investigación Sanitaria (IRYCIS), Madrid, Spain

**Keywords:** HIV, MASLD, NAFLD, gut microbiome, microbiome

## Abstract

**Introduction:**

Metabolic dysfunction-associated steatotic liver disease (MASLD), has emerged as an increasingly recognized problem among people living with HIV (PLWH). The gut-liver axis is considered to be strongly implicated in the pathogenesis of MASLD. We aimed to characterize the gut microbiota composition in PLWH and MASLD and compare it with that of two control groups: PLWH without MASLD and individuals with MASLD without HIV infection.

**Methods:**

We collected clinical data and stool samples from participants. Bacterial 16S rRNA genes were amplified, sequenced, and clustered into operational taxonomic unit. Alpha diversity was studied by Shannon and Simpson indexes. To study how different the gut microbiota composition is between the different groups, beta diversity estimation was evaluated by principal coordinate analysis (PCoA) using Bray-Curtis dissimilarity. To further analyze differences in microbiome composition we performed a linear discriminant analysis (LDA) effect size (LEfSe).

**Results:**

We included 30 HIV^+^MASLD^+^, 30 HIV^+^MASLD^-^ and 20 HIV^-^MASLD^+^ participants. Major butyrate producers, including *Faecalibacterium*, *Ruminococcus*, and *Lachnospira* dominated the microbiota in all three groups. Shannon’s and Simpson’s diversity metrics were higher among MASLD^+^ individuals (Kruskal-Wallis p = 0.047). Beta diversity analysis showed distinct clustering in MASLD^-^, with MASLD^+^ participants overlapping regardless of HIV status (ADONIS significance <0.001). MASLD was associated with increased homogeneity across individuals, in contrast to that observed in the HIV+NAFDL- group, in which the dispersion was higher (Permanova test, p value <0.001; ANOSIM, p value <0.001). MASLD but not HIV determined a different microbiota structure (HIV^+MASLD-^ vs. HIV^+^MASLD^+^, q-value = 0.002; HIV^-^MASLD^+^ vs. HIV^+^MASLD^+^, q-value = 0.930; and HIV^-^MASLD^+^ vs. HIV^+^MASLD^-^, q-value < 0.001). The most abundant genera in MASLD- were *Prevotella, Bacteroides, Dialister, Acidaminococcos, Alloprevotella*, and *Catenibacterium*. In contrast, the most enriched genera in MASLD+ were *Ruminococcus, Streptococcus, Holdemanella, Blautia*, and *Lactobacillus.*

**Conclusions:**

We found a microbiome signature linked to MASLD, which had a greater influence on the overall structure of the gut microbiota than HIV status alone.

## Introduction

Treated HIV is a chronic disease characterized by persistent immune dysfunction, which is affected by the microbiome (1–4), and increased burden of comorbidities, of which non-alcoholic fatty liver disease (MASLD) has emerged as an increasingly recognized problem. Although the intestinal microbiome is implicated in many metabolic conditions (5), little is known about its role in the development of MASLD.

In the general population, MASLD is the most common form of chronic liver disease and might affect more than a quarter of the population worldwide. Its prevalence is steadily increasing, and it is expected to be the leading cause of cirrhosis and hepatocellular carcinoma in the near future (6, 7). In people with HIV (PLWH), liver disease is a leading cause of non-AIDS morbidity and mortality (8). While the impact of hepatitis C virus (HCV) infection is progressively declining, the prevalence and morbidity of MASLD continue to increase in this population (9). Estimates range from 13% to 55%, depending on the population sampled and the diagnostic methods used (6).

The gut-liver axis is considered to be strongly implicated in the pathogenesis of MASLD (10, 11). Changes occurring in the gut microbiome and the host response to the microbiome might contribute to hepatic steatosis, inflammation, and fibrosis. A consistently altered gut microbiota signature is observed when comparing patients with MASLD to healthy individuals as controls, at the level of phylum (increased Proteobacteria), family (increased *Enterobacteriaceae* and decreased *Ruminococcaceae and Rikenellaceae*), and genera (increased *Escherichia*, *Dorea*, *Peptoniphilus* and decreased *Anaerosporobacter*, *Coprococcus*, *Eubacterium*, *Faecalibacterium*, and *Prevotella*) (12). Nevertheless, large discrepancies are found across studies showing divergent results and opposing trends in the abundance of some bacteria (12).

People living with HIV (PLWH) may be at exceptionally high risk for gut-related mechanisms of liver injury due to the reduced diversity of gut microbiome composition described in this population (13). Moreover, alterations in mucosal immunity and increased intestinal permeability lead to translocation of bacterial products and endotoxins to the portal venous system, contributing to liver disease (14, 15). This has been studied in hepatitis B and C virus infection, in which an association between plasma markers indicating impairment of gut epithelial integrity and liver disease progression has been observed (16, 17). Although it is likely that HIV-associated changes in the gut-liver axis further predispose these patients to the development and progression of MASLD, there is no mechanistic study determining the influence of gut microbiota with the progression of MASLD in PLWH.

In this study, we aim to characterize the gut microbiota composition in people with HIV and MASLD (HIV^+^MASLD^+^) and compare it with that of two control groups: PLWH without MASLD (HIV^+^MASLD^-^) and individuals with MASLD without HIV infection (HIV^-^MASLD^+^).

## Methods

### Study design, participants, setting, and eligibility

This is a multicenter prospective cohort study, conducted at the Hospital Universitario Ramón y Cajal and Hospital Universitario la Paz, in Madrid (Spain). From January to December 2018, patients with and without HIV infection who presented hypertransaminasemia maintained in at least two determinations separated by six months were included. Any AST, ALT, or GGT value above the upper limit of normal in our laboratory was considered (**Supplementary Material**). HIV-infected participants were recruited at the HIV clinic, all of whom were on stable ART. HIV-uninfected participants diagnosed with MASLD were recruited at the Metabolic Liver Disease clinic. Comprehensive liver disease assessments, including abdominal ultrasounds and a screening analysis for liver disease (detailed in the **Supplementary Material**), were conducted on all subjects, who were then categorized into MASLD or non-MASLD cohorts. With the results of these tests, an individualized decision was made to perform a liver biopsy according to current guidelines and outside the study protocol. A biopsy was performed on nine participants with a fibrosis score of F3 or higher, confirming steatohepatitis in four of them. Strict exclusion criteria were applied: individuals with active viral hepatitis, significant alcohol (over 30g daily for men, 20g for women) or specific drug abuses (cocaine, heroin, or synthetic drugs), diagnoses of another liver disease (autoimmune, genetic, or drug-induced), decompensated liver disease, hepatocellular carcinoma, recent drug toxicity, or those pregnant or planning pregnancy were disqualified. The research received approval from the Institutional Review Boards of the Carlos III Health Institute in Madrid, Spain (Project PI 17/01717), as well as from the Ethics Committee at the University Hospital Ramón y Cajal (ceic.hrc@salud.madrid.org, Approval Number 097/17). Prior to the commencement of study procedures, all patients provided written informed consent.

### Laboratory methods

#### Sample collection and processing

At the baseline visit, a nutritional survey was conducted (**Supplementary Material**), and a stool sample was collected (having previously given the patient the container for preservation with 95% ethanol). Samples were frozen immediately and stored until processing at −80°C (18).

#### Extraction of DNA from the fecal samples

Fecal samples were thawed at room temperature. Then, DNA was extracted following the procedure described by Lackey et al. (19). Briefly, an aliquot (0.2 g) of each sample was transferred into a sterile tube; subsequently, 0.5 mL of TE50 (10 mM Tris-HCl, 50 mM EDTA, pH 8) were added; after homogenization of the mixture in a vortex, DNA was extracted using the QIAamp^®^ Fast DNA Stool Mini Kit (Qiagen, Germantown, MD), including an initial bead beating step using 0.1 mm diameter zirconia/silica beads (BioSpec Products, Inc., Bartlesville, OK) and a FastPrep FP120A-115 (Qbiogene, Carlsbad, CA). Samples were eluted in 200 μL of the ATE buffer provided in the kit and stored at −80°C until sequencing.

#### Amplification and sequencing of the 16S rRNA gene

A two-step PCR procedure employing dual barcodes was employed to amplify a segment of the V3-V4 hypervariable region within the bacterial 16S ribosomal RNA (rRNA) gene. Universal primers, specifically S-D-Bact-0341-b-S-17 (ACACTGACGACATGGTTCTACACCTACGGGNGGCWGCAG) and S-D-Bact-0785-a-A-21 (TACGGTAGCAGAGACTTGGTCTGACTACHVGGGTATCTAATCC), were utilized at equimolar concentrations. The commercial kit Illumina Microbial Amplicon Library Prep (Illumina, Hayward, CA) was used for library preparation.

Subsequently, Illumina sequencing barcodes were attached to both the 3’ and 5’ ends of the PCR amplicons to enable differentiation between forward and reverse sequences. The concentration of each sample was assessed using a bioanalyzer (2100 Bioanalyzer, Agilent). Afterward, the barcoded PCR products from all samples were combined, targeting approximately equal DNA concentrations, and loaded onto a preparative agarose gel. The correct-sized band was excised, purified using a QIAEX II Gel Extraction Kit (Qiagen), and quantified using PicoGreen (BMG Labtech, Jena, Germany). Finally, one aliquot of the pooled, purified, barcoded DNA amplicons underwent sequencing using the Illumina MiSeq pair-end protocol (Illumina Inc., San Diego, CA, USA) at the Scientific Park of Madrid (Spain). The sequences associated with this study are accessible in the BioSample database of the National Center for Biotechnology Information under BioProject ID PRJNA962007.

#### Statistical methods and bioinformatics analysis

The resulting reads were initially assessed using the *FastQC* software (v0.11.8) and passed a quality control, where the length and quality of the reads were filtered using the *trimmomatic* v0.33 (20) (Paired End method, minimum length of 100, average quality of 30). Outliers were eliminated with *seqkit* v0.11.050 (subcommand *stats*) (21). To normalize for sequencing depth, we used subsampling methods (*seqkit*, subcommand *sample*) based on the minimum number of reads per sample (N = 11429). Amplicon data from the 16S rRNA gene was annotated using the taxonomic sequence classifier *Kraken* (v2.0.8-beta, paired-end option) (22). Taxonomic information on the 16 S rDNA sequences was obtained using the Silva ribosomal RNA Database (23) (release 132) available in the *Kraken* web (24). Taxonomic information of the samples with the abundance data for each Operational Taxonomic Units (OTUs) was used to characterize ecological parameters from the samples. In this study, we focused on analyzing the data at the genus level. The resolution provided by OTUs, together with Kraken2, fit our objectives focused on broader ecological and compositional patterns in the microbiome. This approach allowed us to maintain compatibility with existing literature using OTUs, facilitating direct comparison and integration of our findings. Alpha diversity metrics were computed using the R package *vegan* v2.6-4 (functions *diversity* and *specnumber* for Shannon/Simpson diversity index and observed richness, respectively) (25). Alpha diversity metrics were estimated considering all the taxonomic ranks except for species level. This decision was made because this taxonomy rank is conventionally considered inaccurate (26). Beta diversity was assessed using Bray-Curtis dissimilarity between samples (R package *vegan*, function *vegdist*). Principal Coordinates Analysis (PCoA) of the abundance OTUs data was performed using the built-in R package *ape* version 5.6-2, function *pcoa* (27). For beta diversity measurements and differential abundance analysis, the genus taxonomic level was used. To check if differences between groups composition were significantly different, we used the ADONIS test. A beta-dispersion test with the R package *vegan* (function *betadisper*) together with permanova and ANOSIM test was used for the analysis of multivariate homogeneity of group dispersions. The Tukey HSD multiple comparisons test (function *TukeyHSD*, R package *stats* v4.0.3) was used to test for significance between each group. A Linear discriminant analysis (LDA) effect size (LEfSe) analysis was performed at the genus level to identify the OTUs most likely to explain differences between groups (R package *microbial* v0.0.20, function *ldamarker*). OTUs with LDA scores >4 were plotted in heatmaps (R package *pheatmap* v1.0.12 with a hierarchical clustering in rows based on euclidean distances). Finally, we calculated the Firmicutes/Bacteroidetes ratio for each participant by dividing the relative abundances of Firmicutes by the relative abundance of the Bacteroidetes. We assessed its correlation with quantitative clinical variables, including weight, BMI, waist, and hip circumference, using the Pearson correlation coefficient, and with diagnoses of diabetes and dyslipidemia using regression analysis.

## Results

We included 30 HIV^+^MASLD^+^, 30 HIV^+^MASLD^-^ and 20 HIV^-^MASLD^+^ participants. The characteristics of the study population are provided in **Table 1**. People diagnosed with MASLD have similar characteristics, regardless of their HIV status. These participants were older, had a higher BMI, and had a higher prevalence of comorbidities such as dyslipidemia and diabetes mellitus. Participants with HIV had a higher percentage of men, and all were receiving long-term suppressive antiretroviral therapy for an average of 6 years. The severity of MASLD was comparable in both groups regardless of HIV status, measured by transient elastography (FibroScan). Among participants with MASLD, PLWH had higher alcohol consumption.

**Table 1:** Population baseline characteristics by group.

	MASLD+ HIV- (n=20)	MASLD - HIV+ (n=30)	MASLD + HIV+ (n=30)	p-value
**Age, median (IQR)**	56 (51, 68)	53 (45,56)	54 (43, 59)	0.043
**Male gender, n (%)**	11 (55)	26 (87)	27 (90)	0.011*
Ethnicity, n (%)				
Caucasian	14 (70)	24 (80)	18 (60)	0.218
Latin American	3 (15)	4 (13)	10 (33)	
Sub-Saharan African	0 (0)	1 (3)	0 (0)	
Other/unknown	3 (15)	1 (3)	2 (7)	
**Body mass index (kg/m2), median (IQR)**	31.2 (28.8, 33.8)	24.0 (22.7, 27.1)	27.5 (25.1, 28.7)	0.001*
**Diabetes mellitus, n (%)**	10 (50)	2 (7)	3 (10)	<0.001*
**Dyslipidemia, n (%)**	17 (85)	7 (23)	25 (83)	<0.001**
**Antibiotic use in past 6 months, n (%)**	2 (10)	7 (23)	3 (10)	0.154
Diet (servings per week), median (IQR)				
Legumes	2 (1,2)	2 (0, 3)	2 (1, 3)	0.255
Cereals	7 (7, 7)	21 (8, 26)	7 (7, 7)	0.001**
Vegetables	5 (2, 7)	11 (5, 20)	5 (4, 7)	0.011**
White fish	1 (0, 2)	1 (1, 3)	1 (1, 2)	0.377
Blue fish	1 (1, 2)	1 (1, 2)	1 (1, 2)	0.629
Red meat (beef)	1 (1, 3)	2 (1, 3)	2 (1, 3)	0.669
Pork	2 (1, 3)	2 (1, 4)	1 (1, 2)	0.157
Poultry	3 (2, 3)	2 (1, 3)	3 (2, 5)	0.219
Dairy products	14 (11, 14)	13 (9, 19)	13 (7, 14)	0.338
Fats (oil, butter)	7 (4, 7)	17 (7, 29)	7 (6, 7)	0.001**
Alcohol	0 (0, 1)	0 (0, 2)	3 (1, 7)	0.017
Soft drinks	1 (0, 3)	1 (0, 2)	2 (0, 3)	0.689
MASLD severity				
CAP, median (IQR)	320 (298, 359)	170 (134, 210)	275 (234, 288)	0.004
kPa, median (IQR)	6.8 (5.6, 9.5)	4.4 (3.4, 6.1)	5.3 (4.2, 6.2)	0.002
**Years since HIV diagnosis**, median (IQR)	–	19 (9, 31)	20 (8, 26)	0.460
**Years on ART,** median (IQR)	–	10.1 (7.5, 23.3)	5.9 (5.1, 7.0)	<0.001
**Nadir CD4 T-cell count**, median (IQR)	–	180 (100, 291)	362 (180, 524)	0.003
ART regimen, n (%)				
NNRTI		9 (30)	2 (7)	0.043
PI		3 (10)	1 (3)	
INSTI		15 (50)	25 (83)	
NRTI backbone, n (%)				
TDF/FTC		13 (43)	6 (20)	0.111
TAF/FTC		4 (13)	11 (37)	
ABC/3TC		9 (30)	8 (27)	
Other		4 (13)	6 (17)	
**HCV positive**, n (%)	0 (0)	5 (17)	6 (20)	<0.001*

ALT, alanine aminotransferase; AST, aspartate aminotransferase; ART, antiretroviral therapy; CAP, controlled attenuation parameter; GGT, gamma-glutamyl transferase; IQR, interquartile range; INSTI, integrase strand transfer inhibitor; HCV, hepatitis C virus; HDL, high-density lipoprotein; LDL, low-density lipoprotein, MASLD, metabolic dysfunction-associated steatotic liver disease; NRTI, nucleoside reverse transcriptase inhibitor; NNRTI, nonnucleoside reverse transcriptase inhibitor; PI, protease inhibitor.

All participants with HCV antibodies had undetectable HCV-RNA (cured hepatitis C).

*No statistically significant differences between the two groups of participants with HIV.

**No statistically significant differences between the two groups of participants with MASLD.

**Figure 1:**
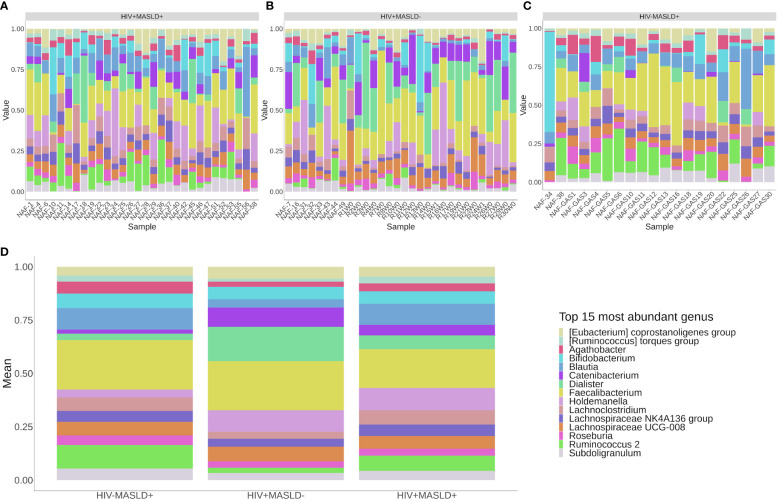
Taxonomic composition of the top 15 most abundant genus. Different study groups HIV+MASLD+, HIV+MASLD- and HIV-MASLD+ are represented in panels **(A**–**C)** respectively, where each bar denote the microbiota composition for each study subject. **(D)** represents the group’s mean.

**Figure 1** shows the taxonomic composition of the top 15 most abundant genus by group. Major butyrate producers, including Faecalibacterium, Ruminococcus, and Lachnospira dominated the microbiota in all three groups.

**Figure 2:**
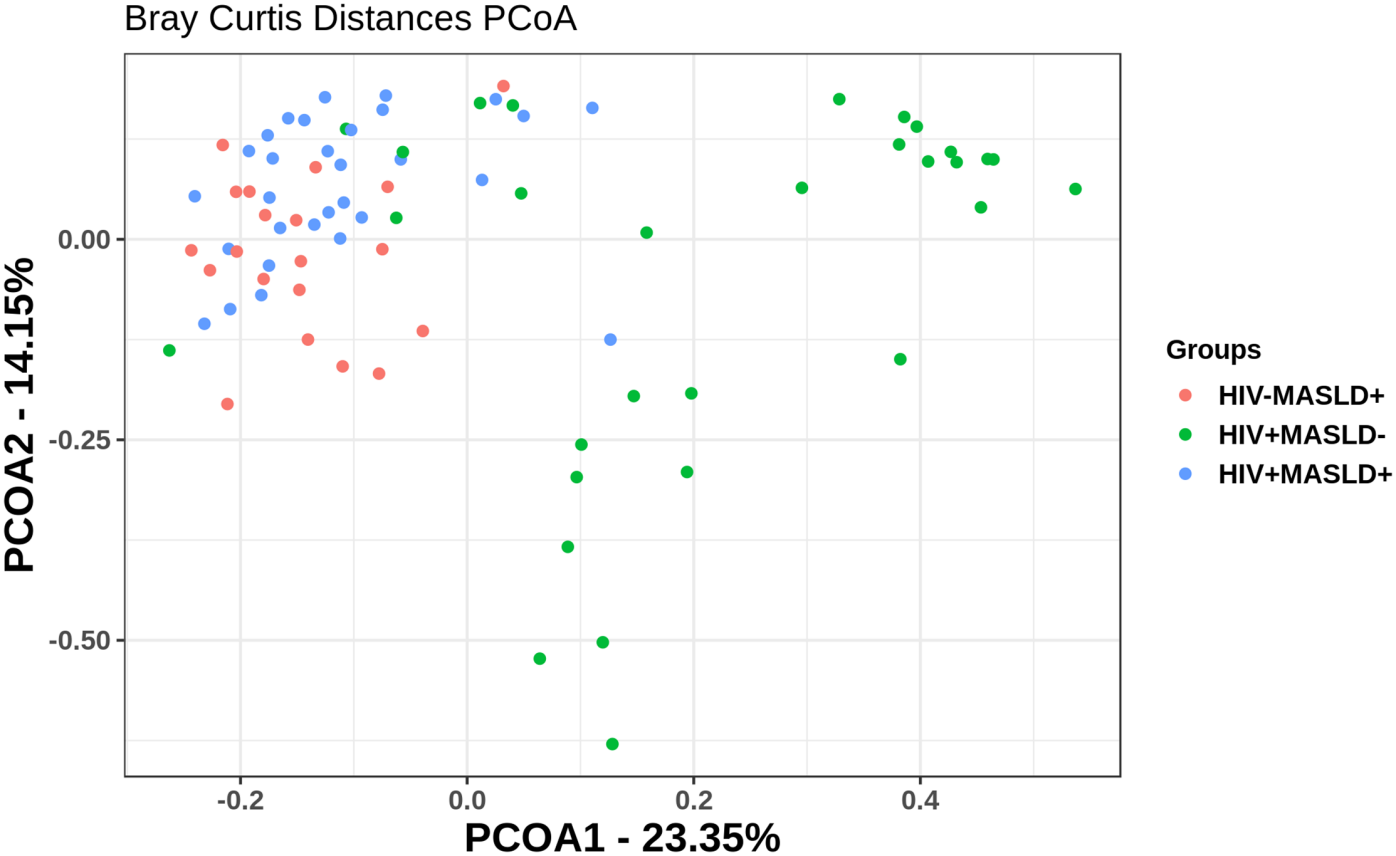
Beta diversity estimation: principal coordinates analysis for Bray-Curtis dissimilarity between samples. Principal coordinate analysis (PCoA) of the Bray-Curtis dissimilarity matrix generated from abundances at the genus level (β-diversity). Each dot represents an individual colored according to the group to which it belongs. The first two dimensions of this PCoA were plotted together with the proportion of variance explained by each of them. (ADONIS significance <0.001).

We used alpha diversity to measure the richness and evenness of bacterial taxa within groups. HIV^+^MASLD^-^ participants had the highest number of observed genus. However, Shannon’s and Simpson’s diversity metrics were higher among MASLD^+^ individuals (Kruskal-Wallis, p = 0.047), indicating that both NAFDL and HIV status had additive effects on alpha diversity (**Supplementary Figure 1**). Beta diversity analysis on Bray-Curtis distances showed distinct clustering in MASLD^-^, with MASLD^+^ participants overlapping regardless of HIV status (**Figure 2**). A permutational analysis of variance (ADONIS) was carried out to evaluate differences in community composition between the groups in our study (HIV^+^MASLD^+^, HIV^+^MASLD^-^, HIV^-^MASLD^+^). The results indicate statistically significant differences in the composition of the communities between the study groups (ADONIS, groups, R² = 0.166, p < 0.001). The sex variable also showed a significant influence, although to a lesser extent (R² = 0.036, p = 0.048), while the age variable did not have a significant effect (R² = 0.011, p = 0.419).

**Figure 3:**
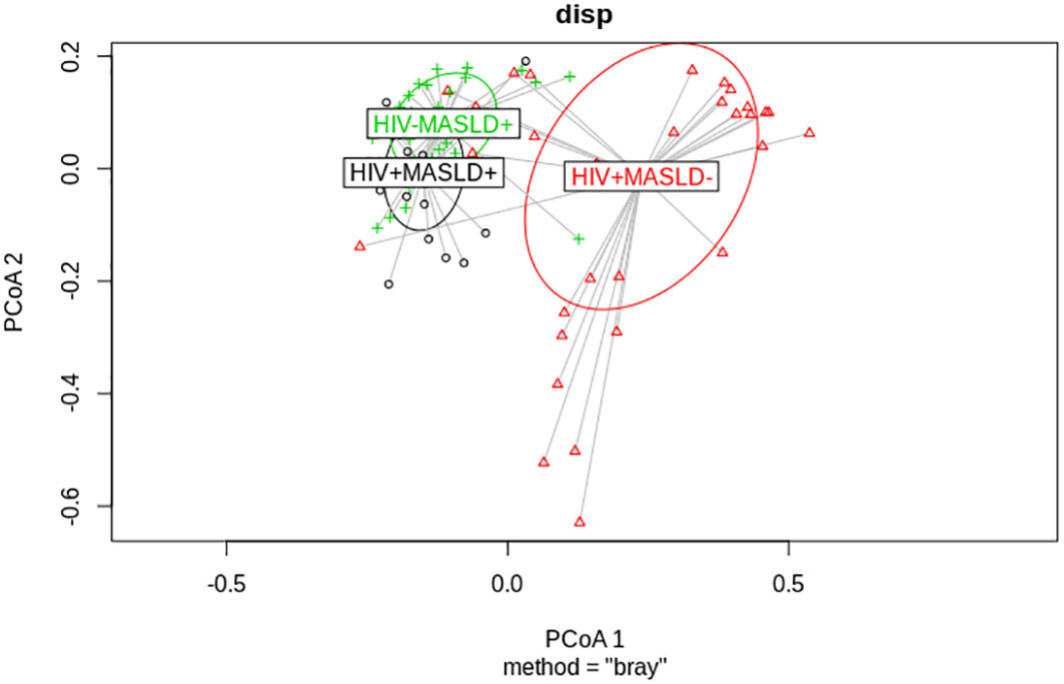
Beta-dispersion plot. Multivariate homogeneity of groups dispersions using Bray-Curtis dissimilarity measures. *Tukey multiple comparisons test*: HIV^-^MASLD^+^ vs. HIV^+^MASLD^+^, q-value = 0.002; HIV^-^MASLD^+^ vs. HIV^+^MASLD^+^, q-value = 0.930; HIV^-^MASLD^+^ vs. HIV^+^MASLD^-^, q-value < 0.001.

Then, we asked whether the within-group beta diversity distances could be affected by the study group. So, we performed beta-dispersion tests. We found that MASLD is associated with increased homogeneity across individuals, in contrast to that observed in the HIV^+^NAFDL^-^ group, in which the dispersion was higher (**Figure 3**, Permanova test, P value <0.001; ANOSIM, P value <0.001). In addition, MASLD but not HIV determined a different microbiota structure (Tukey multiple comparisons test: HIV^+^MASLD^-^ vs. HIV^+^MASLD^+^, q-value = 0.002; HIV^-^MASLD^+^ vs. HIV^+^MASLD^+^, q-value = 0.930; and HIV^-^MASLD^+^ vs. HIV^+^MASLD^-^, q-value < 0.001).

**Figure 4:**
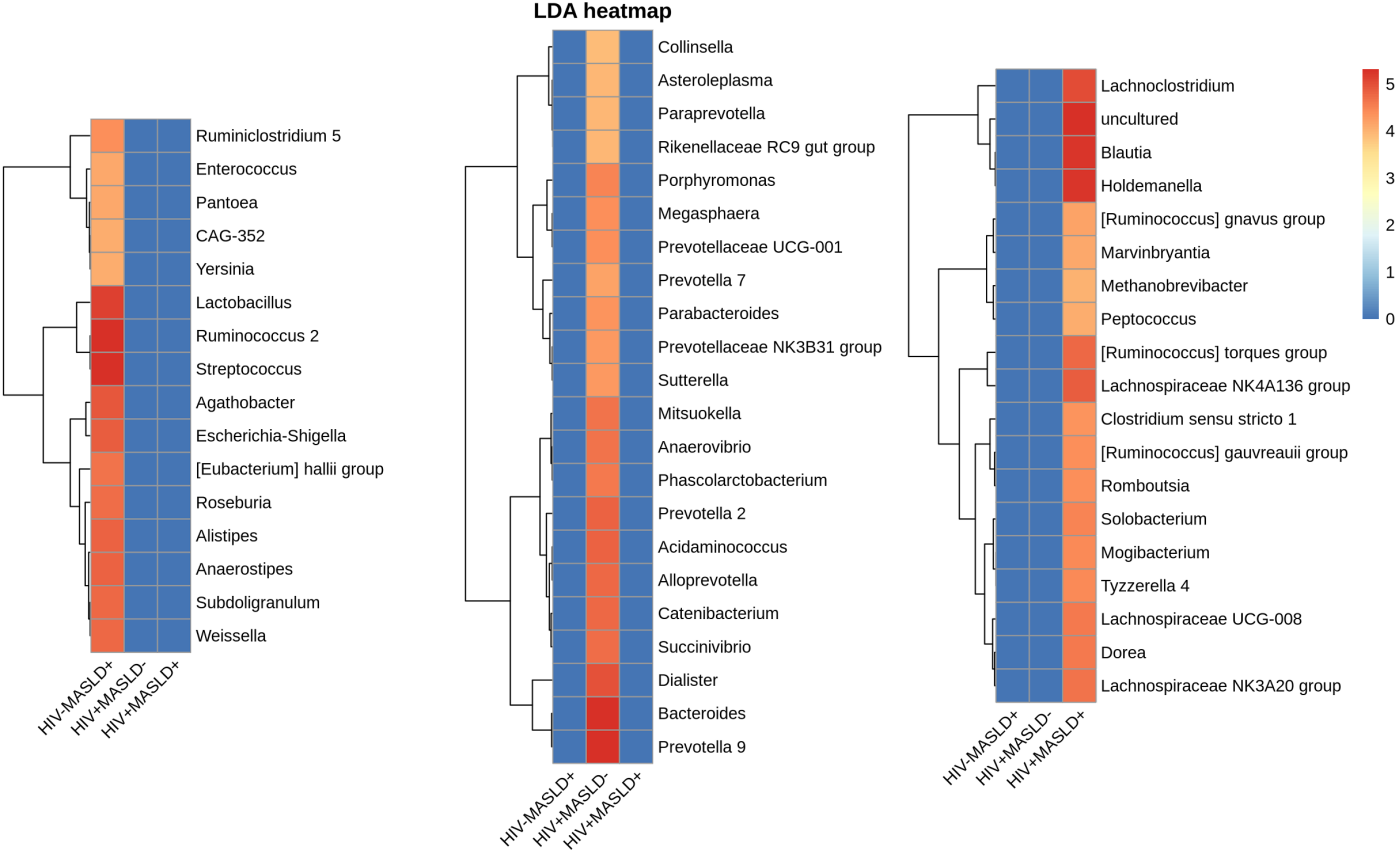
LDA heatmap. Estimated LDA value for those genus (heatmap rows) most likely to explain differences between the three study groups (heatmap columns), being those genus in red the most significant for each group. LDA, Linear discriminant analysis; MASLD, non-alcoholic fatty liver disease.

Subsequently, we investigated which genera determined baseline differences in microbial communities between groups using the LEfSe biomarker discovery tool. **Figure 4** shows the genera most likely to explain differences between groups. Only those genera with a marked difference—whose linear discriminant analysis (LDA) score was greater than 4—have been represented, considering an adjusted p-value threshold < 0.05 in the Kruskal-Wallis test. **Supplementary Figure S2** shows the LEfSe plot. **Supplementary Figure S3** shows the LEfSe plot restricted to participants with HIV, where a similar distribution of genera explaining the difference between participants with and without MASLD is observed.

**Figure 5:**
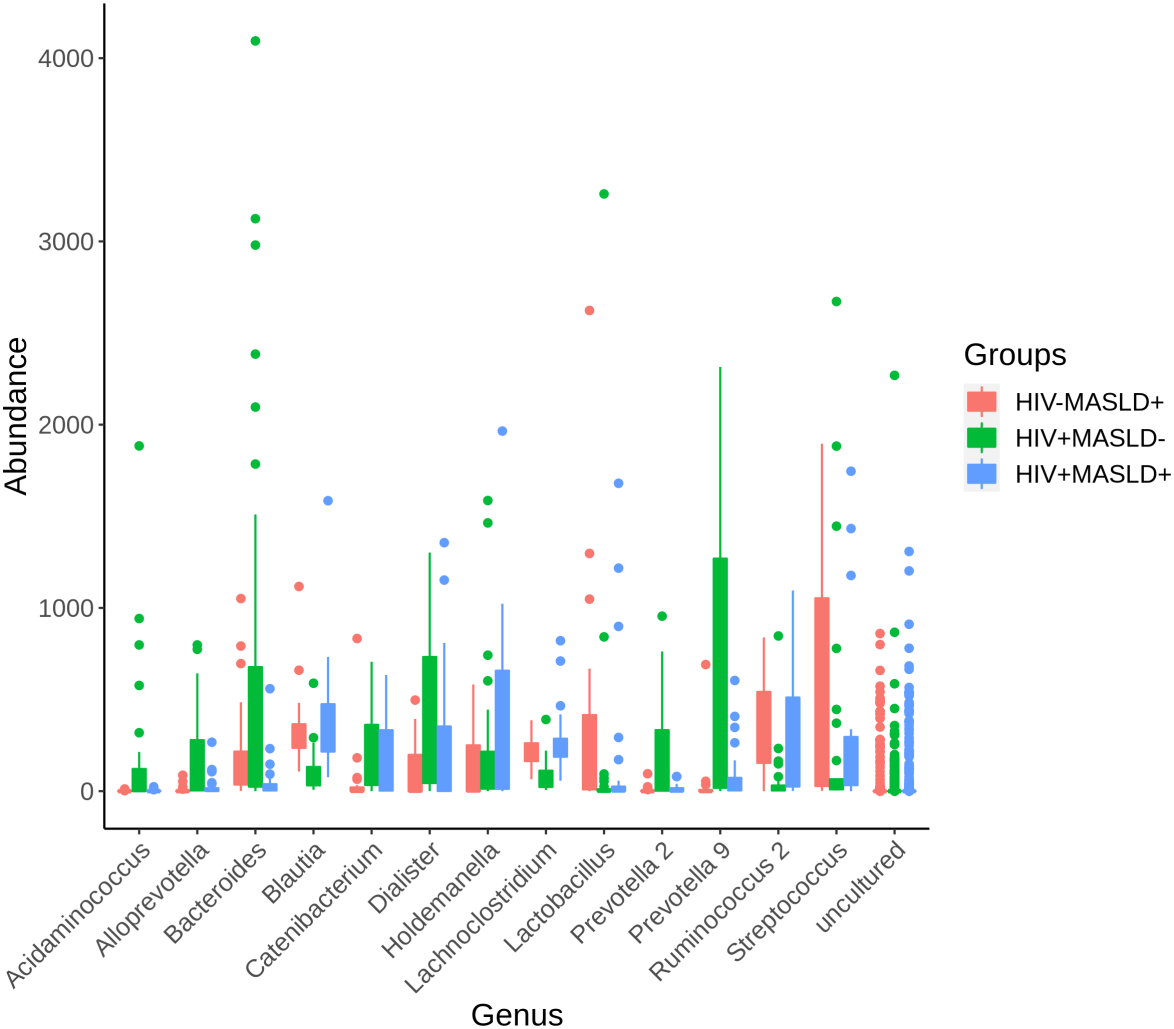
Abundance of the most significant genus according to LEfSe. LefSe analysis examined differences in the microbiome across three groups and selected those with a Linear Discriminant Analysis score >4. Each study group is represented by a color, where a box plot shows the distribution of the most significant genus according to LEfSe. LEfSe, Linear discriminant analysis effect size; MASLD, non-alcoholic fatty liver disease.

In addition, **Figure 5** represents the abundance of the most significant genus according to LEfSe, showing those with LDA > 4. The most abundant genera in participants with MASLD-, and the most likely genera to explain the differences with MASLD+, were *Prevotella, Bacteroides, Dialister, Acidaminococcos, Alloprevotella*, and *Catenibacterium*. In contrast, the most enriched genera in MASLD+ were *Ruminococcus, Streptococcus, Holdemanella, Blautia*, and *Lactobacillus.*

No significant differences were found in the Firmicutes/Bacteroidetes ratio between the three groups of participants (p = 0.203). Correlations with obesity-related variables, mainly BMI, were weak and not significant (r = 0.18, p = 0.195). Likewise, no significant association was found with the presence of diabetes mellitus (p = 0.425) or dyslipidemia (p=0.794).

## Discussion

Our study provides valuable insights into the composition of the intestinal microbiota in individuals with HIV and MASLD, shedding light on the potential implications of the gut-liver axis in the pathogenesis of MASLD.

In contrast to other studies finding a depletion of butyrate producers in PLWH (28), we found that major butyrate producers, including *Faecalibacterium*, *Ruminococcus*, and *Lachnospira*, dominated the microbiota in all three groups (HIV+MASLD+, HIV+MASLD-, and HIV^-^MASLD^+^). This could be explained by socio-environmental factors. Particularly, our study settings included patients in a Mediterranean country, most likely to be under a Mediterranean diet, which seems to promote butyrate-producing bacterial abundance and activity (29, 30). In addition, other factors may play a role, e.g. the fact that all patients had achieved virological suppression (31, 32).

Alpha diversity analysis showed that HIV+MASLD- participants had the highest number of observed genera, indicating a higher richness of bacterial taxa in this group. However, Shannon’s and Simpson’s diversity metrics were higher among MASLD+ individuals, suggesting that both MASLD and HIV status had additive effects on the diversity of the gut microbiota. Although it is often accepted that greater microbiome diversity reflects better health status, there are numerous studies in which microbiota diversity has been found to be greater in PLWH than in healthy controls (33–35). Our findings indicate that the presence of MASLD may influence the microbial composition in PLWH, leading to increased diversity.

Beta diversity analysis revealed distinct clustering in MASLD- participants, while MASLD+ participants overlapped regardless of HIV status. This suggests that the impact of HIV on the gut microbiome is further confounded by MASLD, a highly prevalent condition in PLWH. Although alterations in the microbiome are associated with both the pathogenesis of MASLD and HIV infection, few data exist on the relationship of the gut microbiome and MASLD in PLWH (36). A recent study found similar compositions between PLWH with liver steatosis and controls are similar. However, they showed differences in the bacterial drivers of functional changes in participants with steatosis compared with controls, but not in those with liver fibrosis from other causes. Patients with MASLD had increased abundances of *Eubacterium*, *Finegoldia*, *Faecalibacterium* and *Prevotella*, and decreased abundances of *Akkermansia* and *Bacteroides*, which are associated with functional shifts in bile acid and folate biosynthesis (37). In our study, beta-dispersion tests showed that MASLD is associated with increased homogeneity across individuals, indicating a more consistent microbial community composition in MASLD^+^ participants. In contrast, HIV^+^MASLD^-^ participants exhibited higher dispersion, suggesting greater inter-individual variability in the gut microbiota.

To identify specific genera associated with differences between groups, we performed a LEfSe analysis. Several genera were found to differ significantly between the groups. *Prevotella*, *Bacteroides*, *Dialister*, *Acidaminococcus*, *Alloprevotella*, and *Catenibacterium* were enriched in MASLD^-^ participants and likely contribute to the differences between MASLD^-^ and MASLD^+^ individuals. In contrast, *Ruminococcus*, *Streptococcus*, *Holdemanella*, *Blautia*, and *Lactobacillus* were more abundant in MASLD^+^ participants, suggesting their potential involvement in the pathogenesis of MASLD in PLWH. Although we did not assess the microbiome functions, some mechanisms could explain this microbial signature associated with MASLD. Alterations in bile acid metabolism and signaling have been implicated in MASLD pathogenesis (38). Bile acids are synthesized from cholesterol in the liver and play important roles in fat digestion and regulation of lipid and glucose metabolism. *Blautia* and other gut microbes possess bile salt hydrolase enzymes that deconjugate primary bile acids, altering their signaling properties. Increased levels of *Blautia* and secondary bile acids have been noted in MASLD patients and associated with disease severity (39). Dysregulation of the enterohepatic circulation of bile acids may promote hepatic inflammation and liver fat accumulation. Loss of intestinal barrier integrity and increased gut permeability are also features of MASLD. Certain bacteria enriched in MASLD like *Ruminococcus* and *Streptococcus* may erode tight junctions between enterocytes, enabling translocation of whole bacteria and bacterial products like endotoxin to the liver (40). This can activate inflammatory cascades promoting MASLD progression. Lastly, while specific microbial genes and pathways dysregulated in MASLD are still being unraveled, the altered community likely impacts microbial functions relevant to disease. For example, choline metabolism by gut microbes depletes choline availability which is important for liver function (41). The enrichment of bacteria with increased capacity for dietary lipid metabolism and absorption may also increase calories extracted from the diet worsening fatty liver in people with MASLD (42).

Our study has several strengths, including a well-characterized study population, the use of next-generation sequencing to analyze gut microbiota composition, and the inclusion of two control groups for comparison. However, there are some limitations to consider. First, our sample size was relatively small, which may limit the generalizability of our findings. The inclusion of participants with elevated transaminases could have led to the exclusion of patients with mild steatosis. Our study was cross-sectional, preventing us from establishing a causal relationship between gut microbiota composition and MASLD development or progression in HIV-positive individuals. This work did not include a healthy control group, so no conclusions can be drawn regarding the comparison with this population. Last, we only assessed the microbial composition level. Deeper mechanistic insight is necessary to further understand the effect of these microbes on MASLD pathogenesis.

In conclusion, we found a microbiome signature linked to MASLD, which has a greater influence on the overall structure of the gut microbiota than HIV status alone. We suggest that part of the alterations in the microbiota described as associated with HIV could be confused by the presence of MASLD, which is more prevalent in people with HIV. Future works should address this issue to advance knowledge in this field.

## Data availability statement

The datasets presented in this study can be found in online repositories. The names of the repository/repositories and accession number(s) can be found in the article/**Supplementary Material**.

## Ethics statement

The studies involving humans were approved by Ethics Committee at the University Hospital Ramón y Cajal. The studies were conducted in accordance with the local legislation and institutional requirements. The participants provided their written informed consent to participate in this study. Written informed consent was obtained from the individual(s) for the publication of any potentially identifiable images or data included in this article.

## Author contributions

JM: Formal Analysis, Investigation, Software, Writing – original draft. AT: Formal Analysis, Investigation, Writing – original draft. JD: Supervision, Writing – review & editing. MC: Writing – review & editing. JR: Formal Analysis, Methodology, Software, Writing – review & editing. CA: Investigation, Writing – review & editing. MM: Writing – review & editing. RM: Writing – review & editing. DB: Writing – review & editing. SM: Writing – review & editing. SS: Conceptualization, Methodology, Validation, Writing – review & editing. MS: Conceptualization, Formal Analysis, Investigation, Methodology, Resources, Supervision, Writing – original draft, Writing – review & editing.
