# Supplementary material for: A gut microbiome signature for HIV and metabolic dysfunction-associated steatotic liver disease
Source: Front Immunol. 2023 Dec 14;14:1297378. doi: 10.3389/fimmu.2023.1297378 (PMC10755913; doi:10.3389/fimmu.2023.1297378)
Supplement: Supplementary file 1 [file DataSheet_1.docx]

# **A gut microbiome signature for HIV and metabolic dysfunction-associated steatotic liver disease**

# **Supplementary Material**

# **Table of contents**

Study procedures 2

Supplementary Figure S1 3

Supplementary Figure S2 4

Supplementary Figure S3 5

**Study procedures**

***Inclusion criteria:***

1. HIV-infected patients receiving antiretroviral therapy for at least 1 year with undetectable viremia in the last 6 months (does not apply to comparator population).
2. Alteration of liver biochemistry in two determinations within a 6-month period: elevations of ALT and/or AST and/or GGT and/or AF. Any value above the upper limit of normality of our laboratory will be considered (ALT 40 U/L, AST 50 U/L, GGT 50 U/L, AA 128 U/L)
3. Written informed consent

***Exclusion criteria*:** Participants presenting with:

1. Viral hepatitis.
2. Alcohol abuse (defined by >30 gr daily in men and >20 gr daily in women).
3. Cocaine, heroin, or designer drug abuse.
4. Other known liver diseases: autoimmune, genetic, and drug-related.
5. Isolated alkaline phosphatase alteration.
6. Recent drug toxicity.
7. Impossibility of cannulating a peripheral bloodline in case a liver biopsy is required.
8. Pregnancy, desire for pregnancy.
9. Decompensated liver disease or hepatocarcinoma.
10. Any other comorbidity that, at the investigator's discretion, could prevent correct compliance with the study protocol.

***Procedures***

All participants included underwent an abdominal ultrasound and an analytical screening test for liver diseases, as detailed in the inclusion and exclusion criteria.

Participants not diagnosed with another liver disease by the initial analytical tests underwent non-invasive tests specifically oriented to rule out hepatic steatosis (CAP) and fibrosis (transition elastography, Fibroscan®). With the results of these tests, an individualized decision was made to perform a liver biopsy according to current guidelines and outside the study protocol.


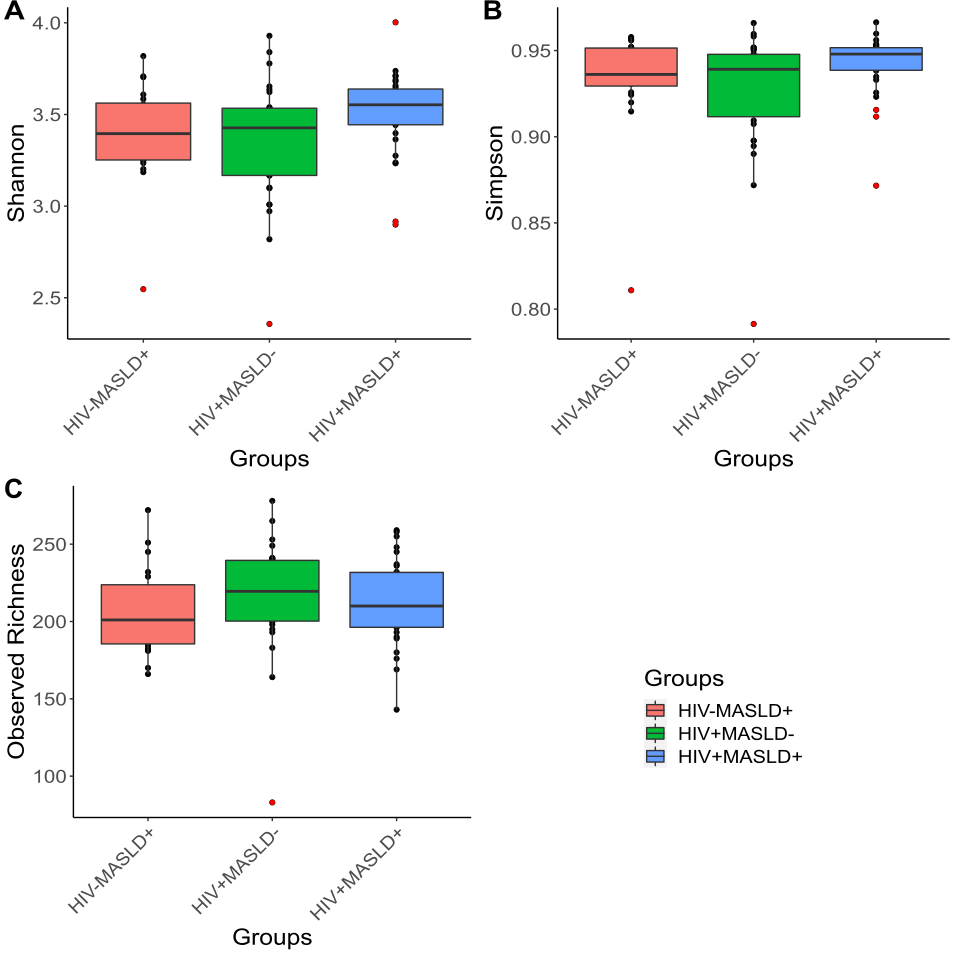


# **Figure S1. Alpha diversity estimation.** **Shannon's and Simpson's diversity metrics and Observed richness. Metrics based on all taxonomic ranks except species.** Boxplots represent the median and quartiles of the alpha diversity estimation for each study group, where each point represents a measure and outliers are highlighted in red.

**
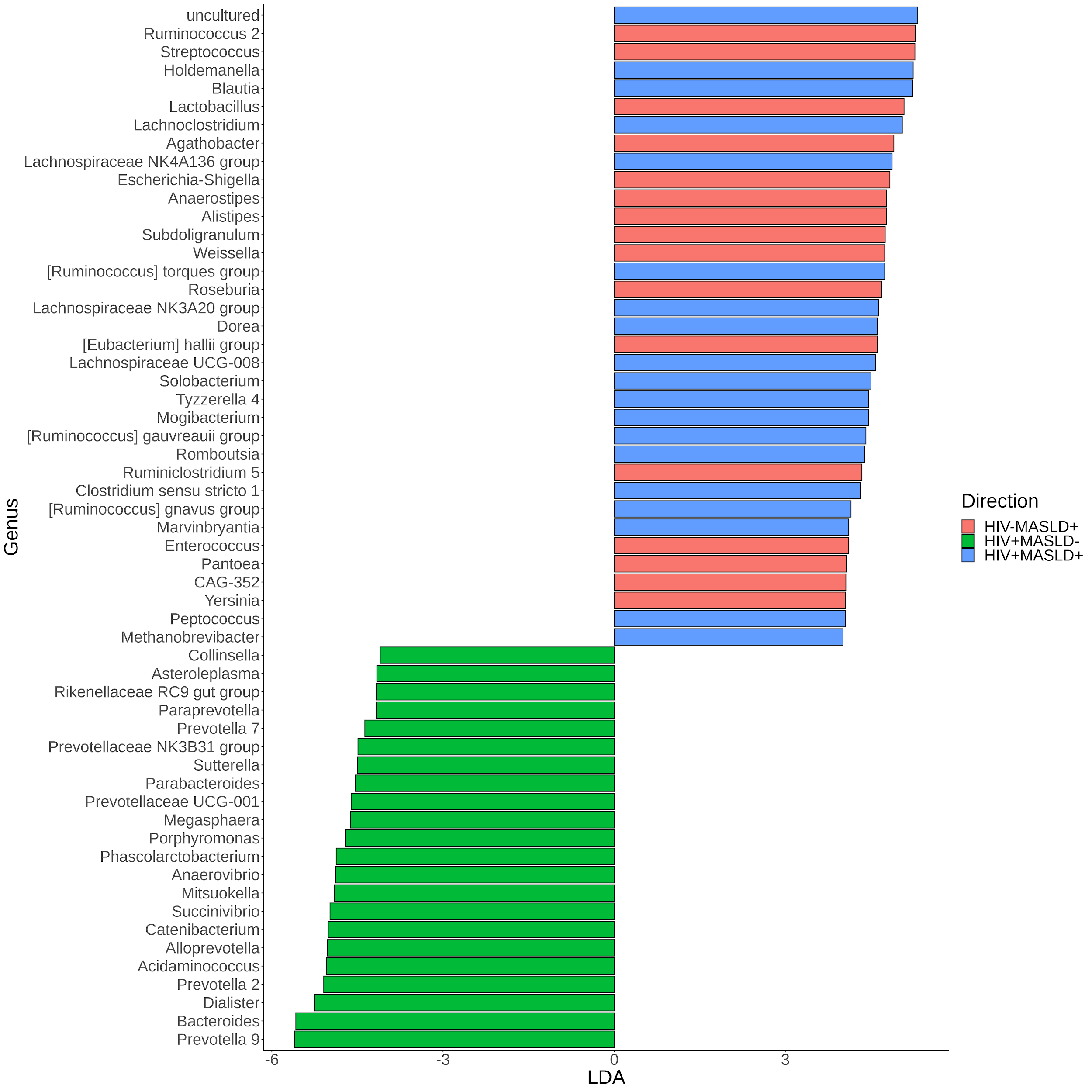
**

**Figure S2. LEfSe plot.** LEfSe analysis graph indicating differentially abundant bacterial genera between the different study groups. Only those genus whose LDA score was greater than 4 have been represented.


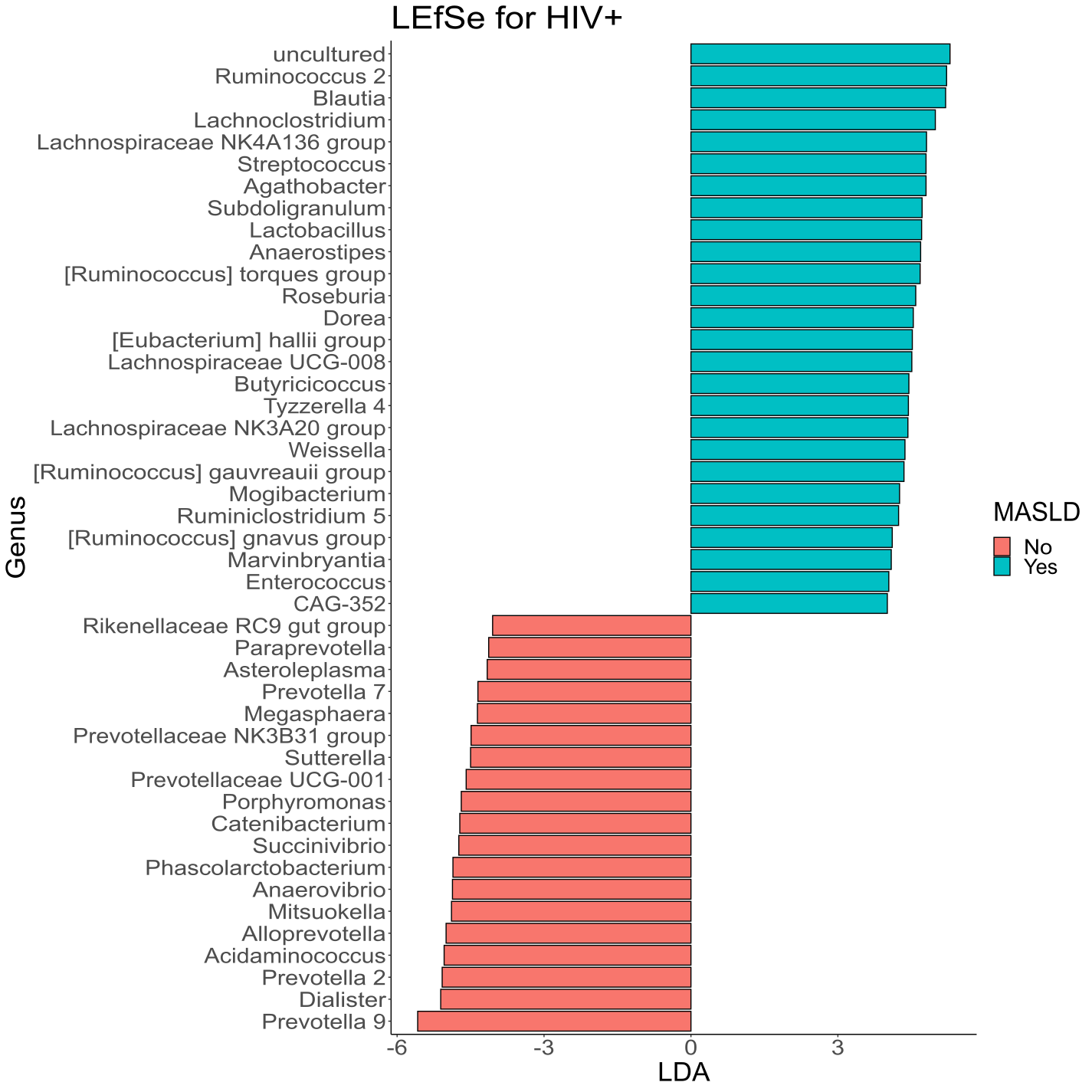


**Figure S3. LEfSe plot for participants with HIV infection.** Estimated LDA value for those genus most likely to explain differences between the presence or absence of MASLD in HIV-infected participants (restricted to LDA >4).

*Abbreviations*: LDA, Linear discriminant analysis; MASLD, metabolic dysfunction-associated steatotic liver disease
